# Cost-effectiveness of midomafetamine-assisted therapy (MDMA-AT) in chronic and treatment-resistant post-traumatic stress disorder of moderate or higher severity: A health-economic model

**DOI:** 10.1371/journal.pone.0313569

**Published:** 2024-11-12

**Authors:** Filip Stanicic, Vladimir Zah, Dimitrije Grbic, Debra De Angelo

**Affiliations:** 1 HEOR Department, ZRx Outcomes Research Inc., Mississauga, Ontario, Canada; 2 Medical Affairs Department, Lykos Therapeutics, San Jose, California, United States of America; Chiba Daigaku, JAPAN

## Abstract

**Objective:**

To explore the cost-effectiveness of midomafetamine-assisted therapy (MDMA-AT) compared to placebo with therapy (PT) in US healthcare settings.

**Methods:**

A health state-transition model was used to analyze the cost-effectiveness of MDMA-AT for treating patients with chronic PTSD of moderate or higher severity. Both treatment arms consisted of 3 preparation (90-min), 3 interventional (8-h), and 9 integration (90-min) sessions, lasting ~4 months total. All sessions included psychotherapy, with interventional also including MDMA or placebo. After receiving treatment, patients were distributed across health states of No PTSD (not meeting PTSD diagnostic criteria), Non-Severe PTSD (treatment responders), Severe PTSD (treatment non-responders), and death. Each state had unique healthcare costs and utilities sourced from real-world data analysis and patient data from MDMA-AT clinical trials (including long-term follow-up). The base-case analysis considered the payer’s perspective with a 5-year horizon, 3.5% annual cost and effect discounts, and an assumed MDMA medication price of $12,000 per session. Trial-derived utilities and US life tables mortality data were used to calculate quality-adjusted life years (QALY). The main outcome was an incremental cost-effectiveness ratio (ICER) with a $150,000 willingness-to-pay (WTP) threshold.

**Results:**

The base-case ICER was $83,845 per QALY. Total direct costs were $64,745 in the MDMA-AT and $33,132 in the PT arms ($31,613 increment). The costs of intervention were $48,376 for MDMA-AT and $12,376 for PT. The highest MDMA medication cost to fit under the WTP threshold was $20,314 per session. Costs related to PTSD healthcare visits and other PTSD treatments were lower with MDMA-AT than PT (-$2,511 and -$1,877 increments, respectively). Utility benefits were higher in MDMA-AT than PT, with 3.691 and 3.314 QALYs generated over 5 years, respectively (0.377 QALY increment).

**Conclusion:**

These data suggest MDMA-AT may be a cost-effective treatment compared to PT for patients with chronic PTSD of moderate or higher severity.

## Introduction

Post-traumatic stress disorder (PTSD) represents a psychiatric syndrome caused by exposure to different types of traumatic events, mostly related to war, sexual abuse, or natural disasters [1]. Studies have shown that the annual PTSD prevalence rate in the US population is up to 9.1% among civilians and 50.2% among military personnel [2]. Not all individuals who are exposed to trauma develop PTSD; onset depends on the nature and severity of trauma and the duration of trauma-related symptoms. However, some population subgroups are more prone to developing PTSD, such as females, those facing childhood adversities, patients with mental health conditions, and individuals with low socioeconomic status or a lack of social support [3]. Chronic PTSD is diagnosed when the traumatized patient fails to recover from a traumatic event and is frequently followed by depression, anxiety, and substance abuse [1, 3].

PTSD is associated with high economic and long-term health outcome burden. The incremental economic burden of US individuals with PTSD compared to those without PTSD in 2018 was estimated at $19.6 thousand per patient. The excess expenditures were also higher among military personnel than civilians ($25.7 thousand and $18.6 thousand per patient, respectively). Additionally, PTSD diagnosis is associated with an increased premature mortality risk of approximately 1.2 times in US civilians and 1.8 times in the US military population [4].

Treatment options for the clinical management of PTSD include psychotherapies and pharmacological interventions [1]. Psychotherapy helps patients cope with and overcome trauma, while medications from different pharmacological classes are used to reduce symptom severity associated with PTSD and other comorbid mental health conditions [1, 5]. Clinical guidelines also consider the augmentation of psychotherapy with pharmacological interventions [6].

However, there are still many challenges associated with PTSD treatments. Available pharmacotherapies used to treat PTSD symptoms typically require long-term use to maintain clinical benefits. This often results in the occurrence of adverse events, leading to treatment discontinuation and worsening symptoms [7]. Additionally, some first-line psychotherapies and pharmacological interventions have shown high treatment failure rates in clinical trials, with many patients retaining PTSD symptoms and frequent dropout [8].

Midomafetamine used in combination with psychological intervention (MDMA-AT) is an investigational treatment approach being evaluated by Lykos Therapeutics for adult patients with PTSD. Although the exact mechanism of action is unclear, it is hypothesized that the synergistic effects of combined midomafetamine and manualized psychotherapy treatment more directly impact pathological PTSD mechanisms rather than only suppressing the symptoms. By affecting the serotoninergic activity in the left amygdala, midomafetamine is thought to alter the trauma processing in PTSD patients by attenuating fear, reducing reactions to stress triggers, eliminating chronic hyperarousal, and decreasing defensiveness without blocking access to memories or preventing a deep and genuine experience of emotion. The effects of midomafetamine is therefore proposed to augment manualized psychotherapy during MDMA-AT sessions [9, 10]. Phase III randomized clinical trials (RCTs) reported significant efficacy of investigational MDMA-AT compared to placebo with therapy in terms of reducing symptom severity in participants with chronic PTSD of moderate or higher severity. The treatment course consisted of only three MDMA sessions in combination with psychological intervention and close patient observations. Adverse events that occurred were typically transient, mild to moderate severity, and promptly managed [11, 12].

The challenges of current PTSD treatments suggest the need for novel clinical interventions to treat patients. MDMA-AT demonstrates the potential to address this unmet need in the current PTSD treatment landscape. Although efficacy and safety were shown in clinical trials, the cost-effectiveness of this intervention remains unclear. This health economic model aims to explore the cost-effectiveness of the MDMA-AT for treating patients with chronic PTSD of moderate or higher severity in the US healthcare setting.

## Methodology

Model development and reporting of findings are aligned with the updated Consolidated Health Economic Evaluation Reporting Standards (CHEERS) checklist [13]. The research is based on the principles of mathematical modeling, published peer-reviewed literature, publicly available US statistics, and de-identified clinical trial data. The study does not require ethics board approval as there are no relevant or potential conflicts with ethical principles in medicine and research.

Clinical trial data supporting the study findings were used under license from Lykos Therapeutics and are publicly unavailable. Data access may be granted by the authors upon reasonable request and gained approval from Lykos Therapeutics. Any data that might be shared would be released via a data use agreement.

The model development and cost-effectiveness analysis were performed using Microsoft Office Excel® software (version Office 365). Clinical trial data were analyzed using the IBM SPSS Statistics® software (version 23.0).

### Model design

A health state transition economic model with a cost-effectiveness analysis was developed from the payer perspective. The modeling time frame was 25 years with implemented annual cycles. The model population included patients with chronic PTSD of moderate or higher disease severity. The treatment included three interventional MDMA-AT sessions as well as preparation and integration psychotherapy sessions based on clinical trial protocols [9, 11, 12]. Briefly, participants received three 90-minute preparation psychotherapy sessions prior to the first MDMA-AT session. Each interventional MDMA-AT session lasted 8 hours and consisted of MDMA administration, psychotherapy, and close patient monitoring by two trained therapists. MDMA was administered as a split dose of 80 mg and 40 mg (120 mg total) during the first MDMA-AT session and as a split dose of 120 mg and 60 mg (180 mg total) in the second and third sessions. MDMA-AT treatment sessions were spaced approximately four weeks apart followed by three weekly 90-minute integration sessions comprised of psychotherapy alone with two therapists. For all preparation, interventional, and integration sessions, a manualized psychotherapy approach was used. Manualized psychotherapy is inner-directed and designed to invite inquiry and facilitate a therapeutic effect by providing support for approaching difficult material that does not interfere with the participant’s spontaneous experience.

The model comparator was identical to the protocol used in the MDMA-AT arm including manualized psychotherapy, but patients were administered placebo instead of MDMA during the interventional sessions.

The modeling was based on data from MAPP1 and MAPP2 studies–two randomized, double-blind, phase III clinical trials comparing MDMA-AT to placebo with therapy administration in participants with chronic PTSD of moderate or higher disease severity [11, 12]. After completing the phase III trials, some participants were included in the long-term follow-up study (MPLONG) which was used to estimate the durability of treatment effects.

Model health states were No PTSD, Non-severe PTSD, Severe PTSD, and death. At model initiation, all patients had moderate or higher severity PTSD; therefore, they were classified in the Non-severe or Severe PTSD health states. After receiving treatment with MDMA-AT or placebo with therapy (4-month post-treatment endpoint), patients transitioned to other health states based on the treatment response:

- **No PTSD**–patients who responded to the treatment and experienced loss of PTSD diagnosis
- **Non-severe PTSD–**patients with severe PTSD with treatment response but without loss of PTSD diagnosis (still meet the criteria for PTSD) and patients with moderate PTSD who did not experience loss of the PTSD diagnosis (regardless of treatment response)
- **Severe PTSD–**severe PTSD patients who did not respond to treatment and those that withdrew from treatment
- **Death–**based on US mortality of the general population with the same age as the average age of MAPP1 and MAPP2 participants [14] and death events from clinical trials (MAPP1, MAPP2, MPLONG).

Loss of PTSD diagnosis in clinical trials was defined as not meeting PTSD diagnostic criteria after treatment according to the Diagnostic and Statistical Manual of Mental Disorders, Fifth Edition (DSM-5) and measured using the Clinician-Administered PTSD Scale (CAPS-5). Treatment response was defined as a clinically relevant CAPS-5 score decrease of at least 10 points. As such, more effective treatment would be depicted by a higher rate of patients in the No PTSD state, while less effective treatment would show higher rates of patients with Non-severe and Severe PTSD.

The durability of treatment effects was assessed in the follow-up MPLONG study of MAPP1 and MAPP2 participants. Data from this long-term observational study were analyzed to obtain transitioning rates between the model states at the MPLONG endpoint by applying the previously explained criteria. The mean follow-up duration in the MPLONG trial was 17 months for participants with moderate or higher severity PTSD. Patient distribution among health states at the 1-year endpoint was estimated using distribution data at trial enrollment, the 4-month post-treatment endpoint, and the 17-month long-term follow-up endpoint. The proportion of patients in the No PTSD state treated with MDMA-AT at these endpoints was graphically represented with the proportion of sample as the y-axis and time in months as the x-axis. Using the best-fit method (log model), proportions were extrapolated over time and extracted for a 1-year time point. The difference between the proportions of patients in the No PTSD state at the estimated time point and a previous time point was equally distributed between the Non-severe and Severe PTSD states. The patient distribution at the 1-year time point was employed for the first annual cycle, while distribution of patients extracted from the MPLONG trial was used for all other consecutive model cycles (from 2^nd^ annual cycle to the maximum 25^th^ annual cycle) due to the lack of data in the literature. The same method was used to estimate transitioning rates between the model health states among patients treated with the placebo with therapy comparator.

**Fig 1:**
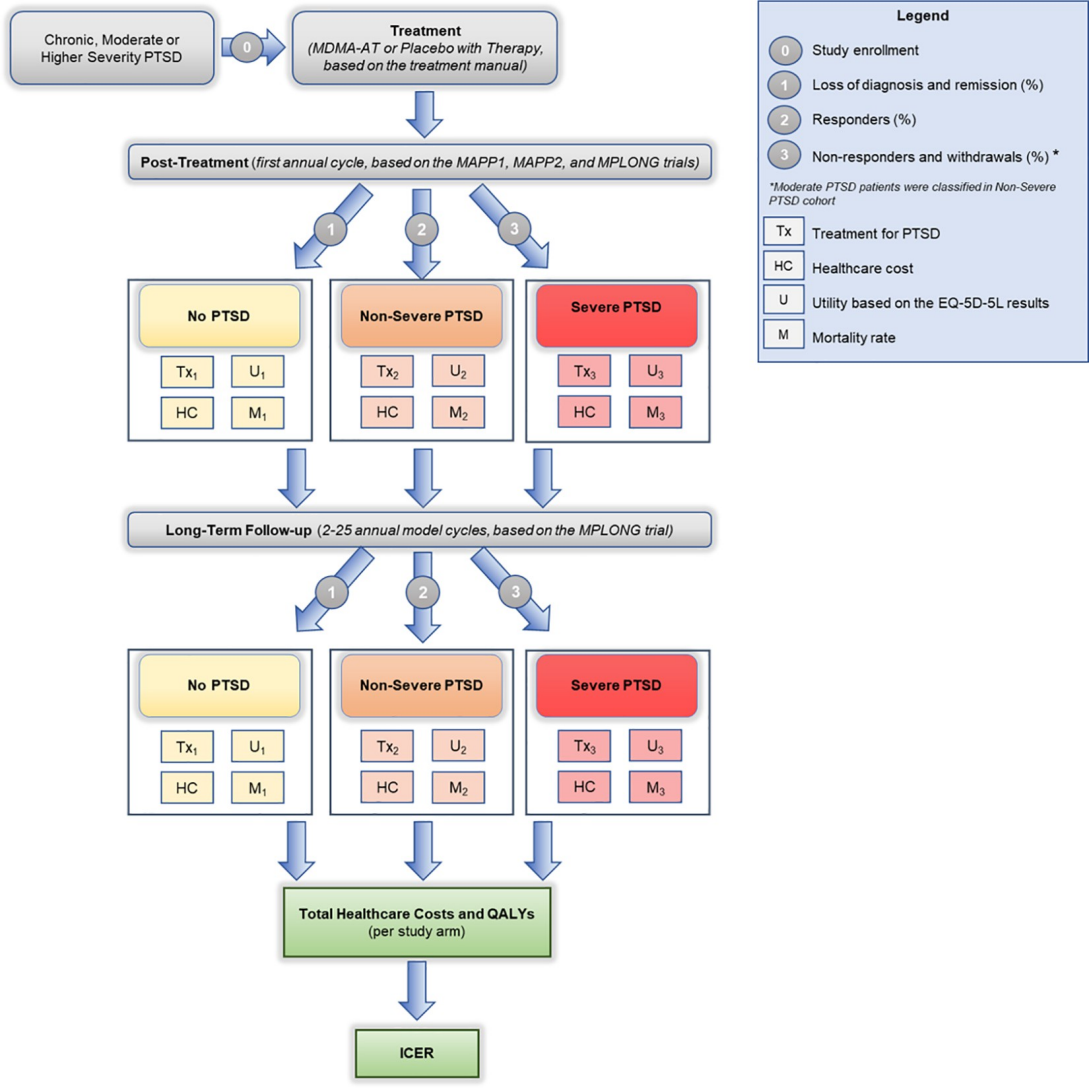
Model scheme diagram. Abbreviations: PTSD–Post-traumatic stress disorder; MDMA-AT–MDMA-assisted therapy; EQ-5D-5L - European quality of life—5 dimension—5 level; PT–placebo with therapy; QALY–Quality-adjusted life years; ICER–Incremental cost-effectiveness ratio.

Briefly, the model started when the target population initiated the treatment with MDMA-AT or the placebo with therapy comparator. After the treatment course (4 months for the MAPP1 and MAPP2 trials), patients were stratified into one of the health states (No PTSD, Non-severe PTSD, and Severe PTSD). Each state had its own PTSD treatment costs, healthcare visit costs, utilities, and mortality rates. Afterwards, patients were observed over the long-term follow-up (MPLONG trial) and redistributed across health states based on the changes in their health status. Healthcare costs, utilities, and incremental cost-effectiveness ratio (ICER) at relevant endpoints were calculated. The full model scheme diagram is shown in Fig 1.

### Model parametrization

Each health state was characterized by unique healthcare costs and utility inputs. Healthcare cost inputs were pooled from a PTSD burden of illness (BOI) study based on real-world evidence data. The cohorts in the BOI analysis match the model health states (No PTSD = Baseline PTSD, Non-severe PTSD = PTSD without comorbidities, and Severe PTSD = PTSD with comorbidities). PTSD economic burden and treatment characteristics in the BOI analysis were assessed during the 2-year follow-up period [15].

Healthcare costs included three components: intervention treatment costs (MDMA-AT or placebo with therapy), healthcare visits for PTSD, and regular PTSD treatments (psychotherapy or medication). Intervention costs were one-time expenditures in the model paid for MDMA-AT or placebo with therapy. According to the clinical trial protocols [11, 12], there were three interventional sessions (MDMA or placebo administration with 8-hour psychotherapy) with a total of nine integration and three preparation 90-minute psychotherapy sessions provided by two therapists. Hence, the MDMA-AT intervention cost was the sum of three preparation psychotherapy sessions, three MDMA-AT sessions (which included the estimated MDMA medication price plus eight hours of psychotherapy), and nine integration psychotherapy sessions. The comparator intervention cost included only expenditures related to the psychotherapy components (three preparation, three placebo with therapy, and nine integration sessions). The cost of a single preparation or integration session was pooled from the BOI analysis using an average 90-minute psychotherapy session cost multiplied by two (the number of therapists per session). The cost of a single 8-hour session (480 minutes) was estimated from a 90-minute psychotherapy cost input by multiplying with a time coefficient (480 minutes divided by 90 minutes) and the number of therapists (2 therapists).

The second cost component represents healthcare expenditures associated with PTSD services provided in outpatient, inpatient, and emergency department (ED) settings. PTSD-related visit costs per cohort from BOI analysis were used to estimate the average monthly cost of PTSD-related healthcare services per model health state. To calculate the PTSD visit costs for MDMA-AT and the placebo with therapy comparator, the monthly PTSD visit costs per health state were multiplied by the rate of patients in each health state that received respective intervention treatment and summed per model arm. The average monthly PTSD visit costs were multiplied by the annual rate (12, for the total number of months) to estimate PTSD visit expenditures per model cycle, and then multiplied by model endpoint duration (i.e., 1-year, 5-year, 10-year…) to determine the total PTSD visit costs.

The final component of healthcare costs was regular PTSD treatment expenditures including psychotherapy, FDA-approved medications, and off-label medications. Psychotherapy utilization rates and the number of sessions per month were extracted from the MPLONG data. The monthly psychotherapy cost per model state was calculated using monthly utilization data from MPLONG and the average cost of psychotherapy sessions from the BOI analysis. Medication utilization rates were not available from the MPLONG data and were therefore determined from BOI analysis. The monthly medication expenditures (FDA-approved and off-label separately) were estimated using the utilization rates per cohort and 2-year costs related to the medication group from BOI analysis. The 2-year medication group costs were divided by 24 months to estimate a mean cost on a monthly level. The monthly PTSD treatment costs for model health states were the sum of monthly psychotherapy, FDA-approved medication, and off-label medication expenditures per respective state. The same method as for the PTSD visit costs was applied to calculate monthly PTSD treatment costs per model arm (MDMA-AT or placebo with therapy) using the results per health state. Since other PTSD treatments were not allowed during the MAPP1 and MAPP2 trials, PTSD treatment costs were not included in the calculation while patients were receiving MDMA-AT or placebo with therapy sessions (until the end of the 4th month in the model).

Health-related utility values were measured with the European Quality of Life—5 Dimension—5 Level (EQ-5D-5L) tool in MAPP1, MAPP2, and MPLONG trials. Using the post-treatment and long-term follow-up results for each health state per study arm, the 1-year values were estimated by applying the previously explained modeling method. The utility values for MDMA-AT were derived by summarizing the multiplied results of patient distribution across health states in the intervention arm and utility values per each health state in the same arm. The calculation was also applied to gain utility estimates for the placebo with therapy comparator at all endpoints.

The base-case analysis included patients with PTSD of moderate or higher disease severity, the economic burden from the payer’s perspective, a 5-year modeling time horizon, and 3.5% annual discount rates on cost and utility inputs. A brief description of all model input parameters and relevant data sources for base-case analysis are presented in Table 1.

**Table 1:** Base-case analysis variable values and the relevant data sources.

Variable	MDMA-AT value	Placebo with therapy value	Input source
**Patient distribution (%) for the first annual cycle**			
No PTSD	79.39%	43.28%	Extrapolated results using log curve function based on MAPP1, MAPP2, and MPLONG clinical trial data analysis
Non-severe PTSD	8.67%	20.37%	
Severe PTSD	11.70%	36.12%	
**Patient distribution (%) for all other annual cycles (2–25 years)**			
No PTSD	82.89%	45.34%	Extracted results from the MPLONG clinical trial data analysis
Non-severe PTSD	9.83%	21.16%	
Severe PTSD	7.02%	33.25%	
5-year cumulative mortality rate	1.32%	1.32%	Extracted data from US life tables [14] and death events from MAPP1, MAPP2, and MPLONG clinical trial data analysis
Age on baseline	39 years		Extracted from MAPP1 and MAPP2 clinical trial data analysis
**Healthcare costs (USD)**			
Preparation	$442	$442	Estimated from real-world data analysis findings (BOI study)
8-hour psychotherapy	$2,357	$2,357	Estimated from real-world data analysis findings (BOI study)
MDMA administration	$12,000	$0	Assumption
Integration session	$442	$442	Estimated from real-world data analysis findings (BOI study)
Monthly PTSD visits			
No PTSD	$70	$70	Extracted from real-world data analysis (BOI study)
Non-severe PTSD	$112	$112	
Severe	$226	$226	
Monthly PTSD treatment			
No PTSD	$203	$203	Extracted from real-world data analysis (BOI study) and MPLONG clinical trial data analysis (psychotherapy utilization)
Non-severe PTSD	$338	$338	
Severe	$283	$283	
**Utility (EQ-5D-5L scores) for the first annual cycle**			
No PTSD	0.816101	0.791415	Extrapolated results using log curve function based on MAPP1, MAPP2, and MPLONG clinical trial data analysis
Non-severe PTSD	0.710804	0.711006	
Severe PTSD	0.619287	0.596565	
**Utility (EQ-5D-5L scores) for all other annual cycles (2–25 years)**			
No PTSD	0.821930	0.790370	Extracted results from the MPLONG clinical trial data analysis
Non-severe PTSD	0.714290	0.729170	
Severe PTSD	0.612000	0.603180	

Abbreviations: MDMA-AT–MDMA-assisted therapy; PTSD–Post-traumatic stress disorder; EQ-5D-5L –European quality of life—5 dimension—5 level

### Outcomes and sensitivity analyses

Discount rates of 3.5% per year of modeling were applied to cost and utility inputs. The pre-defined willingness-to-pay (WTP) threshold was $150,000. ICER was the main study outcome, calculated as the ratio of healthcare costs increment and quality-adjusted life years (QALY) increment between MDMA-AT and psychotherapy.


$$ICER=\frac{{Cost}_{MDMA-AT}-{Cost}_{PT}}{{QALY}_{MDMA-AT}-{QALY}_{PT}}$$

Abbreviations: ICER–Incremental cost-effectiveness ratio; MDMA-AT–MDMA-assisted therapy; PT–placebo with therapy; QALY–Quality-adjusted life years

Univariate one-way sensitivity analysis (OWSA) was implemented in the model to denote which input parameters had the greatest impact on study findings. By varying input values in a specified range (±10%), the impact of each parameter was presented in a tornado chart.

Probabilistic sensitivity analysis (PSA) assessed the reliability of study findings by showing the impact of uncertainty regarding model inputs. By running a high number of iterations (varying input values 5,000 times according to the assigned data distribution), the results were reported as the ICER of mean cost increment and mean QALY increment, a scatter plot of all cost and QALY increments (each dot representing the result of a single simulation), and a cost-effectiveness acceptability curve.

Scenario analysis with a goal-seek function was also implemented in the model. This analysis calculated the maximum MDMA-AT session cost at which the ICER is equal to the WTP threshold. It also provided ICER values for decreasing MDMA-AT cost per session estimate.

## Results

The base-case analysis ICER (patients with PTSD of moderate and higher severity, payer perspective, 5-year horizon, and 3.5% annual cost and utility discounts) is $83,845 per QALY. MDMA-AT is a cost-effective treatment compared to the placebo with therapy comparator in the base-case setting.

The cost of MDMA-AT treatment was $48,376 ($36,000 for MDMA and $12,376 for all psychotherapy components of MDMA-AT), with a $16,125 price for a single MDMA-AT session ($12,000 for MDMA, $2,357 for 8-hour psychotherapy, and $1,768 for preparation and integration sessions). The total cost of the placebo with therapy was $12,376.

**Fig 2:**
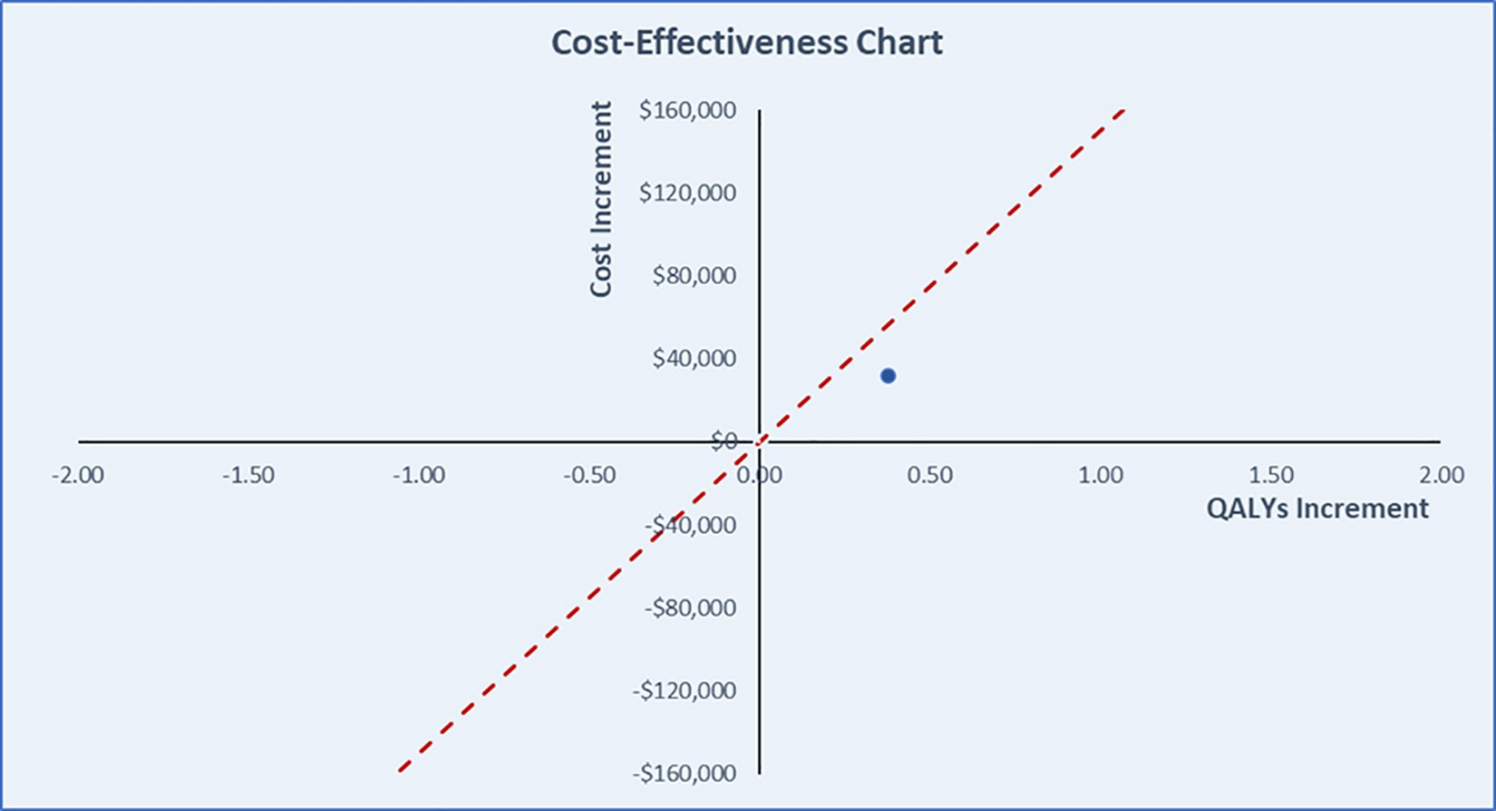
Cost-effectiveness chart of base-case findings with a standard WTP threshold.

Total direct PTSD costs over a 5-year time horizon were $64,745 for the MDMA-AT arm and $33,132 for the placebo with therapy arm ($31,612 incremental cost). The cost difference was mainly driven by the intervention costs, with a much higher cost in MDMA-AT than in placebo with therapy ($48,376 and $12,376, respectively). However, MDMA-AT had lower costs related to PTSD visits ($4,831 compared to $7,342 in placebo with therapy) and PTSD treatment expenditures ($11,538 compared to $13,414 in placebo with therapy). Unlike the healthcare costs, utility outcomes were more beneficial for MDMA-AT than placebo with therapy (3.691 QALY vs. 3.314 QALY, respectively). The cost-effectiveness chart using the base-case scenario and $150,000 WTP threshold is presented in Fig 2.

**Fig 3:**
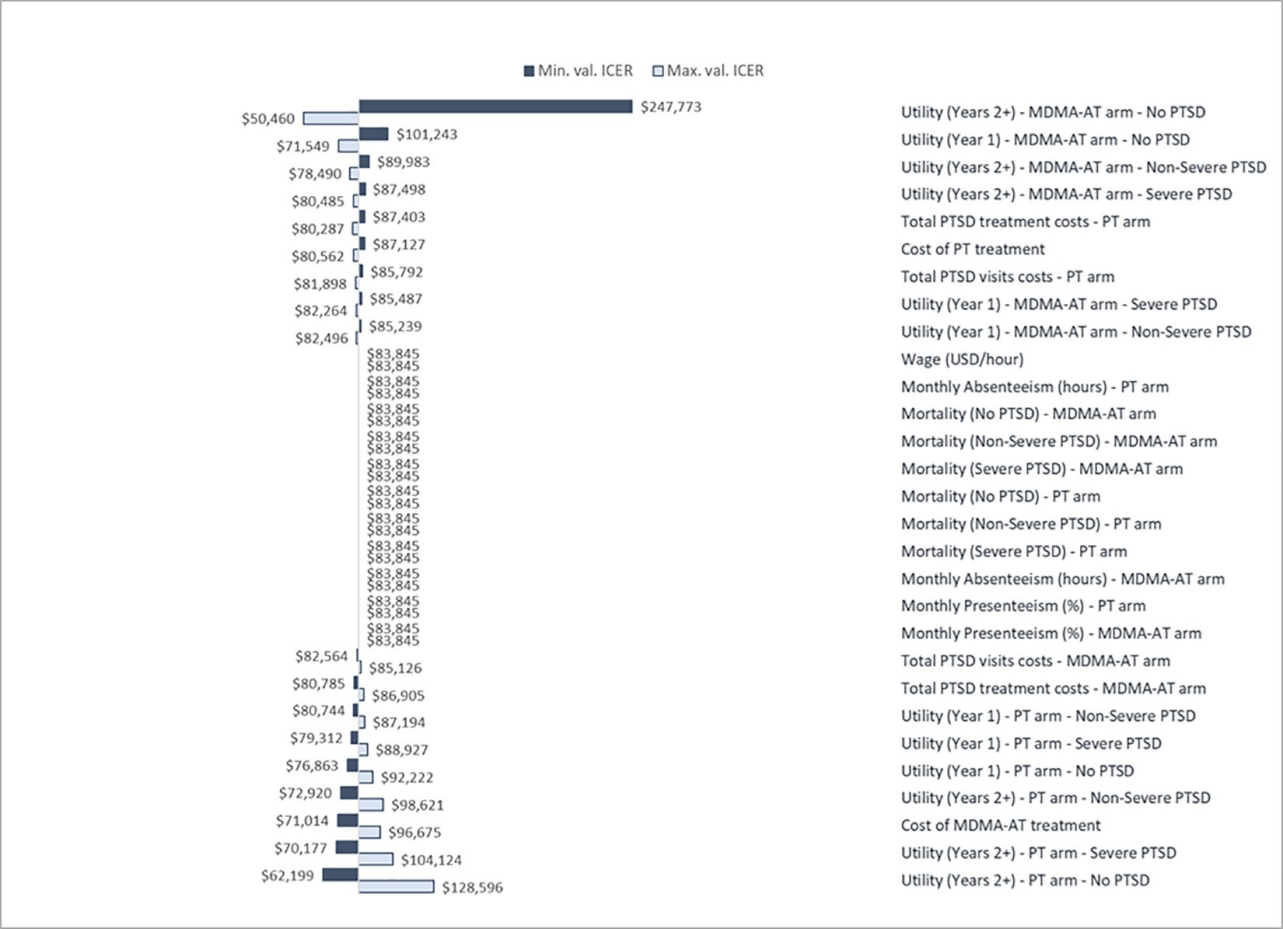
Tornado diagram with 10% input variations using univariate OWSA. Abbreviations: ICER–incremental cost-effectiveness ratio; MDMA-AT–MDMA-assisted therapy; PT–placebo with therapy; PTSD–Post-traumatic stress disorder.

OWSA with a 10% variation rate denoted utility inputs as parameters with the highest impact on findings. Lowering MDMA-AT utility in the No PTSD category for 2–25 model cycles by 10% yielded an ICER of $247,773 per QALY, while increasing the value by 10% provided an ICER of $50,460 per QALY. After varying MDMA-AT utility in the No PTSD category for the first model cycle by +10% or -10%, ICER values were $71,549 and $101,243 per QALY, respectively. Univariate OWSA findings are presented in Fig 3.

**Fig 4:**
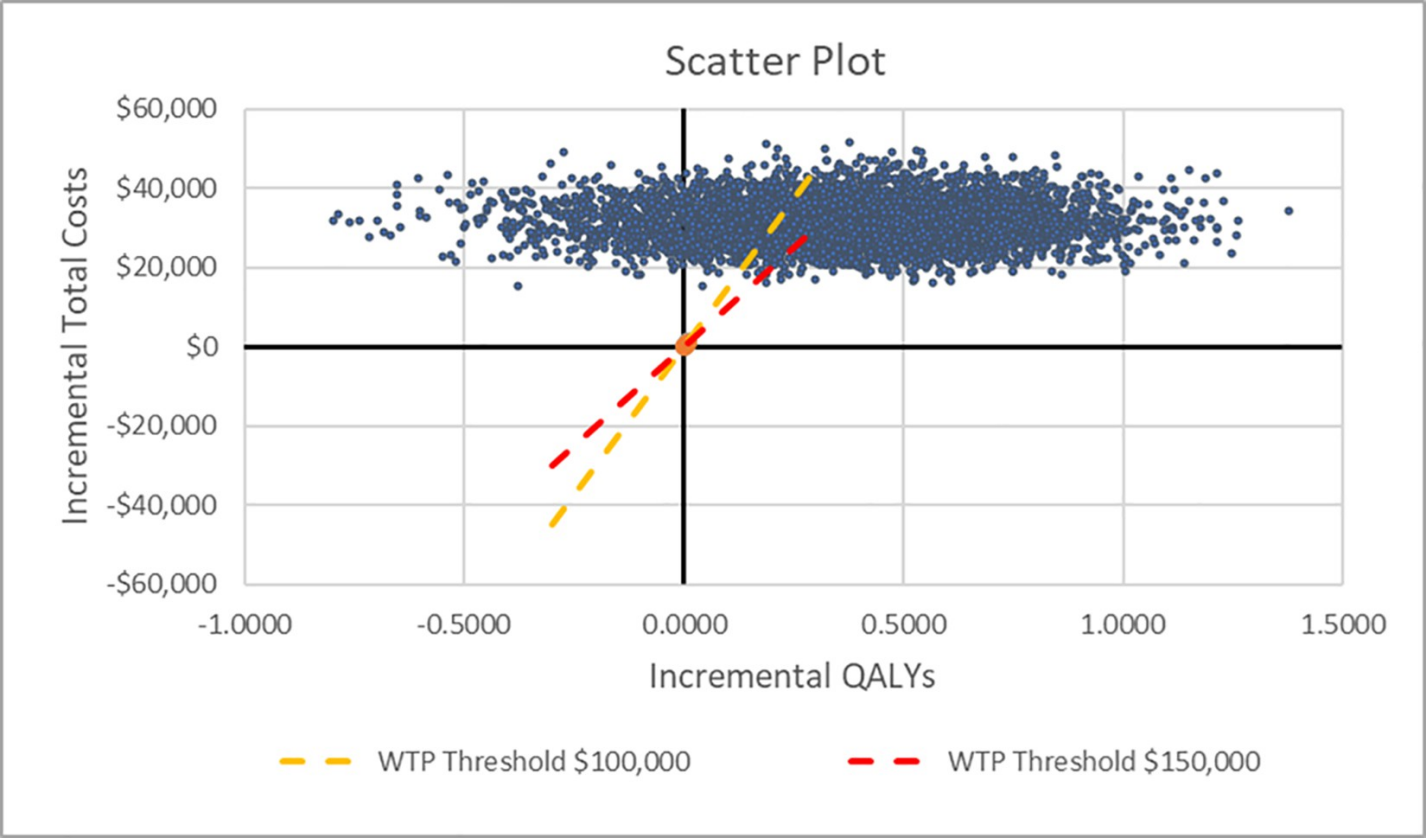
Scatter plot for base-case analysis. Abbreviations: QALY–Quality-adjusted life years; WTP–Willingness-to-pay.

**Fig 5:**
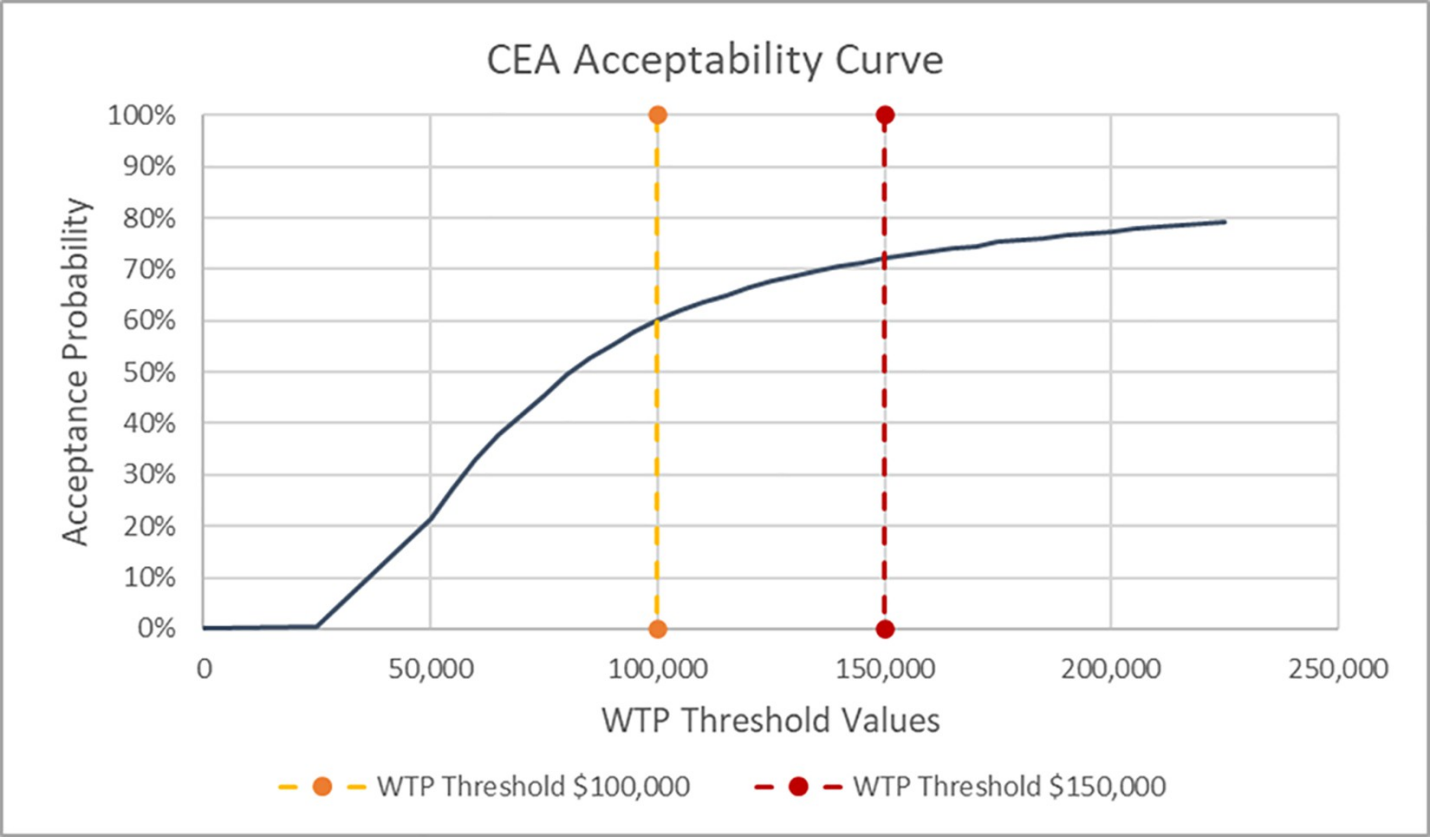
Cost-effectiveness acceptability curve for base-case analysis. Abbreviations: WTP–Willingness-to-pay.

PSA was performed with 5,000 iterations. The mean direct PTSD expenditures for base-case analysis were $64,805 in the MDMA-AT arm and $33,106 in the placebo with therapy arm, yielding a mean cost increment of $31,699 (±5,378 standard deviation). The average QALY increment was 0.376 (±0.305 standard deviation) with mean utilities of 3.691 in the MDMA-AT arm and 3.315 in the placebo with therapy arm. The estimated ICER using the mean increments was $84,240 per QALY. These findings were consistent with the main results, establishing the cost-effectiveness superiority of MDMA-AT over the placebo with therapy comparator. The acceptability of MDMA-AT compared to placebo with therapy was 72.12% at the $150,000 WTP threshold and 60.16% at the $100,000 WTP threshold. PSA findings are presented as scatter plot (Fig 4) and cost-effectiveness acceptability diagrams (Fig 5).

Scenario analysis calculated the maximum MDMA medication cost per session with the base-case analysis ICER equal to the pre-defined WTP threshold. It was demonstrated that a maximum MDMA medication price of $20,314 per session yielded an ICER of $149,998 per QALY, meeting a WTP threshold of $150,000. Besides the maximum price, scenario analysis provided an overview of ICER value distribution at lower MDMA medication costs. The maximum MDMA medication price to fit under the $100,000 WTP threshold was $14,030 per session, while $7,746 per session was a maximum cost for WTP threshold of $50,000.

## Discussion

This cost-effectiveness analysis estimated the healthcare costs per QALY in patients with chronic PTSD of moderate or higher severity, comparing MDMA-AT and placebo with therapy. Model inputs were based on data from three clinical trials (two phase III and one long-term follow-up), a real-world economic BOI analysis, and treatment patterns in modern clinical practice to minimize assumptions and provide the most accurate price estimate. The analysis yielded a cost of $48,376 for the MDMA-AT treatment course, including three preparation 90-minute sessions ($442 per session), three interventional sessions consisting of MDMA medication administration ($12,000 per session) and 8-hour psychotherapy ($2,357 per session), and nine 90-minute integration psychotherapy sessions ($442 per session). Using these cost estimates, the reported ICER was $83,845 per QALY. This was considered cost-effective as the WTP value in the model was $150,000 per QALY. The threshold was defined by the World Health Organization recommendation stating that the cost-effective boundary should be between one and three times the Gross Domestic Product (GDP) per capita [16]. The World Bank data for 2022 reported $76,329.58 GDP per capita for the US [17], yielding a cost-effective threshold range between $76,330 and $228,989 per QALY, which corresponds to the MDMA medication cost of $11,056 to$30,242 per session. However, the authors used a median value (approximately $150,000) to have a fair comparison and considered it relevant for the modern US healthcare system. Regarding sensitivity analyses, univariate OWSA (10% parameter variation) identified utility changes due to MDMA-AT treatment as the most impactful parameter on study findings. The results were also consistent with 5,000 simulations in PSA. The ICER was lower than the WTP threshold in 72.12% of iterations.

There are several published cost-effectiveness analyses including MDMA-AT for PTSD treatment. The most recent was published in 2022 by Marseille et al., including phase III clinical trial findings. Although the model had a different design than this study and was based on the Markov principles, it also demonstrated the cost-effectiveness of MDMA-AT over the standard of care comparator with ICER of $47,554 per QALY at 1-year follow-up. In addition, standard of care was dominated by MDMA-AT at 10- and 30-year periods after receiving the intervention. The cost of full-course MDMA-AT treatment was $11,537 per patient [18]. However, the Marseille et al. model was based on multiple assumptions due to a lack of real-world economic and treatment patterns data. The authors used only MAPP1 clinical trial data as MAPP2 and MPLONG trials were not available at the time. Therefore, the Marseille et al. model was developed for patients with chronic severe PTSD, excluding those with moderate disease severity. Also, the long-term follow-up estimate may not be reliable since the phase III trial followed patients for only 4 months, and the model horizon covered a 30-year period after MDMA-AT administration. Another Marseille et al. economic model with an identical design used the findings of six phase 2 clinical trials in which MDMA-AT showed superiority to the standard of care arm among chronic, severe, treatment-resistant PTSD patients. MDMA-AT was cost-effective at 1-year endpoint (ICER $26,427 per QALY) and dominated standard of care at 10- and 30-year periods. The estimated price of two MDMA-AT (which included two interventional sessions) was $7,543 per patient. However, the model was based on small sample trials in patients with severe and treatment-resistant PTSD. Although the findings from a long-term assessment of phase II trials completers were incorporated in the model (mean follow-up 45.4 months), it included only 19 patients, which significantly limited the generalizability of results on 1,000 patients for the 30-year period [19]. Unlike the previous MDMA-AT health economic models, the inputs in this study were purely based on the real-world evidence and data of two phase III clinical trials, including a long-term observation of a larger sample of participants, to provide the best possible estimate. Despite the model covering 25-year period, primary findings presented benefits for a shorter time horizon (5 years), ensuring a higher level of reliability since the average length of follow-up in MPLONG from the parent trial baseline was 17 months. In addition, the long-term phase II clinical trial conducted by Mithoefer et al. demonstrated sustainable effects of MDMA-AT during an average of 3.8-year follow-up period, without significant changes in CAPS scores compared to baseline [20].

Mavranezouli et al. performed a network meta-analysis using available literature sources, supplemented by expert opinion, to populate a health economic model that compared 10 PTSD interventions with no treatment from the UK healthcare setting perspective [21]. The authors pointed out substantial between-study heterogeneity, mainly originating from the differences in PTSD patient characteristics and included interventions, which significantly affect the reliability of the findings. The results imply that Eye Movement Desensitization and Reprocessing (EMDR) was the most cost-effective PTSD intervention, with a 1.8 QALY increment and £33,928 net monetary benefit (NMB) per patient. EMDR was followed by combined somatic/cognitive therapies (£33,364 NMB), self-help with support (£32,880 NMB), and psychoeducation (£32,754 NMB). The most cost-effective medication treatment compared to no treatment was the use of a selective serotonin reuptake inhibitor (SSRI) with an NMB of £32,316 [21].

A systematic literature review by von der Warth et al. captured all economic evaluations of PTSD patients published until February 2020 regardless of the sample population, traumatic experience, or intervention type. The findings denoted that most published studies were low-to-moderate quality, lacked relevant reported information, and had inappropriate designs. Trauma-focused cognitive-behavioral therapy (TF-CBT) was the most assessed treatment with an ICER of $13,162 per QALY gained compared to usual treatment and $39,366 per additional PTSD remission compared to breathing retraining and psychoeducation comparator. Authors identified the need for accurate and comprehensive economic evaluations of PTSD interventions [22]. Hence, our study addresses this unmet need in the published literature by providing precise estimates of PTSD economic burden and extensive reporting of model findings.

The health economic analyses of medications in PTSD are lacking in the published literature, with limited studies that evaluate cost-effectiveness of pharmacological treatments for PTSD. Mihalopoulos et al. [23] compared SSRIs and trauma-focused CBT to standard treatment practice for adults with PTSD in the Australian healthcare setting. The median ICER for trauma-focused CBT was $19,000 per QALY, with 100% uncertainty iterations falling beneath a $50,000 per QALY threshold. SSRIs showed a much lower ICER of only $200 per QALY, most likely due to a very low price, but the results were highly uncertain [23]. Another cost-effectiveness analysis by Le et al. [24] compared prolonged exposure psychotherapy to sertraline in a double-randomized treatment preference trial. Prolonged exposure psychotherapy was dominant to sertraline treatment with lower healthcare costs (-$262) and more QALY gained (0.056). The probability of cost-effectiveness with a $100,000 per QALY threshold was 93.2% [24].

### Strengths and limitations

Unlike other health economic models, this study provides highly precise estimates by utilizing real-world economic BOI and treatment patterns data of patients with PTSD instead of previously published literature. Analyzing individual patient data from clinical trials instead of using aggregated published results also increased the reliability of model findings. However, as with other health economic models, the study has several limitations that should be considered while interpreting the results.

The main limitation refers to the BOI study and clinical trials used to populate the model inputs. Any limitations regarding inputs from these sources directly apply to this economic evaluation as well. The second limitation is the generalizability of model findings. Although the model used data from two clinical trials and a long-term follow up study, the total number of patients may not be sufficient to fully reflect the real-world sample. Also, heterogeneity in population characteristics (i.e., comorbidities, disease severity, demographics) may limit the generalizability. Third, the choice of the model comparator was limited due to a lack of evidence on direct comparisons and specific study protocols. Hence, the comparator arm was the placebo with therapy from MDMA-AT clinical trials instead of treatments from PTSD clinical practice. Fourth, the average observation of patients in the follow-up study was 17 months, while the model time horizon was 25 years.

## Conclusion

This study demonstrated that MDMA-AT was a more cost-effective treatment than placebo with therapy for patients with chronic moderate or higher severity PTSD. Inputs with the highest impact on model findings were quality of life benefits in treatment and comparator arms. The results were consistent with 5,000 simulations.

Based on these findings, MDMA-AT is a novel option for patients with chronic PTSD that may decrease disease burden, including economic aspects, and improve patient outcomes after a single treatment course.
